# Psychometric validation of the Cannabis Withdrawal Checklist in a Spanish sample with cannabis use disorder

**DOI:** 10.3389/fpsyg.2026.1744004

**Published:** 2026-05-15

**Authors:** Judith Saura, Ariadna Feliu, Marta Enríquez, Xavier Roca, Montse Ballbè, Marcela Fu, Lidia Segura, Esteve Fernández, Cristina Martínez

**Affiliations:** 1 Tobacco Control Unit, Cancer Control and Prevention Program, WHO Collaborating Center on Tobacco Control, Institut Català d’Oncologia – ICO, l’Hospitalet de Llobregat, Barcelona, Spain; 2 Cancer Control and Prevention Group, Institut d’Investigació Biomèdica de Bellvitge – IDIBELL, l’Hospitalet de Llobregat, Barcelona, Spain; 3 CIBER en Enfermedades Respiratorias – CIBERES, Instituto de Salud Carlos III, Madrid, Spain; 4 Department of Clinical Sciences, School of Medicine and Health Sciences, Universitat de Barcelona, Barcelona, Spain; 5 Department of Primary Care and Public Health, School of Public Health, Imperial College London, London, United Kingdom; 6 Addictive Behaviors Unit, Department of Psychiatry, Hospital de la Santa Creu i Sant Pau, Barcelona, Spain; 7 Institut d'Investigació Biomèdica Sant Pau (IIB Sant Pau), Barcelona, Spain; 8 Addictions Unit, Department of Psychiatry, Institute of Neurosciences, Hospital Clínic de Barcelona – IDIBAPS, Barcelona, Spain; 9 Department of Public Health, Mental Health, and Maternal and Child Health Nursing, School of Nursing – Bellvitge Campus, Universitat de Barcelona - UB, Barcelona, Spain; 10 Secretariat of Public Health, Department of Health, Generalitat de Catalunya, Barcelona, Spain; 11 Philip R. Lee Institute for Health Policy Studies, University of California San Francisco, San Francisco, CA, United States

**Keywords:** Cannabis withdrawal, cannabis-tobacco co-use, psychometric assessment, scale validation, substance use disorders

## Abstract

**Introduction:**

Cannabis is used globally. In Spain, it is often used in combination with tobacco, which may exacerbate withdrawal severity. Currently, no validated instrument exists to assess cannabis withdrawal symptoms in Spanish populations. This study aimed to validate the 15-item Cannabis (Marijuana) Withdrawal Checklist (CWC) for Spanish-speaking individuals who co-use cannabis and tobacco.

**Materials and methods:**

This psychometric validation study was nested within a multicenter longitudinal project conducted across 10 substance use treatment programs in Catalonia (Spain). The CWC was forward- and back-translated following COnsensus-based Standards for the selection of health Measurement INstruments (COSMIN) recommendations. Internal consistency, construct validity, and sensitivity to change over time were assessed using Cronbach’s alpha, exploratory factor analysis (EFA), and mixed-effects analysis. Data from 82 participants were used to conduct the validation.

**Results:**

The adapted CWC demonstrated high internal consistency (Cronbach’s *α* = 0.918 at baseline; 0.885 at 2 weeks of treatment). EFA supported a unidimensional structure explaining approximately 60% of the variance. Although symptom severity did not significantly change over follow-up, withdrawal intensity was positively associated with the level of cannabis use. The scale effectively captured clinically relevant symptoms such as irritability, craving, and sleep difficulties.

**Conclusion:**

The Spanish CWC showed strong reliability and acceptable psychometric properties, supporting its use for assessing cannabis withdrawal symptoms in Spanish-speaking adults, particularly those who co-use tobacco products. The scale may be valuable in both research and clinical settings, particularly for monitoring withdrawal during treatment interventions. Further validation using larger, more diverse samples is warranted.

**Clinical trial registration:**

Clinicaltrials.gov, identifier NCT0551209.

## Highlights

- We adapted and validated the Marijuana Withdrawal Checklist for a Spanish population.
- The new Cannabis Withdrawal Checklist demonstrated good psychometric properties.
- Withdrawal severity was strongly correlated with cannabis use intensity.
- Common cannabis–tobacco co-use underscores the scale’s real-world applicability.
- The adapted scale is suitable for monitoring cannabis withdrawal symptoms.

## Introduction

Cannabis is the third most commonly used drug worldwide (Connor et al., 2021). In Europe, approximately 8% of adults (22.6 million people aged 15–64 years) reported cannabis use in 2024 (EMCDDA (European Monitoring Centre for Drugs and Drug Addiction), 2024). In Spain, the prevalence of use is similar: in 2022, 10.5% of individuals aged 15–64 years reported cannabis use within the past year and 8.6% within the past month (OEDA (Observatorio Español de las Drogas y las Adicciones), 2023). These figures highlight the substantial public health relevance of cannabis use and the need to address related health consequences, including cannabis use disorder (CUD).

According to the Diagnostic and Statistical Manual of Mental Disorders, Fifth Edition (DSM-5), CUD is defined as a problematic pattern of cannabis use leading to clinically significant impairment or distress, characterized by the presence of at least two of the 11 diagnostic criteria within a 12-month period (APA, 2014). One of these criteria is cannabis withdrawal syndrome, which typically occurs after abrupt cessation or reduction of cannabis use following heavy and prolonged use. Screening tools such as the Cannabis Abuse Screening Test (CAST), a six-item instrument widely used in epidemiological research, can help identify individuals at risk of CUD (Legleye et al., 2007).

Cannabis withdrawal symptoms typically emerge within 24–72 h after cessation or reduction of use following heavy and frequent use (Connor et al., 2021). Common symptoms include irritability, anxiety, sleep disturbances, reduced appetite, restlessness, physical discomfort, fatigue, and concentration difficulties (APA, 2014). Symptoms generally peak within the first week and subside within 2 weeks, though sleep-related disturbances may persist longer (Connor et al., 2021; Budney and Hughes, 2006). Because withdrawal symptoms are strongly associated with relapse and difficulties in maintaining abstinence, their systematic assessment is clinically relevant (Allsop et al., 2012).

In Spain, cannabis is commonly consumed in combination with tobacco, a pattern that may influence both use trajectories and withdrawal experiences (Peters et al., 2012; Tullis et al., 2003). Co-use has been associated with greater health risks, including anxiety and depression, cognitive impairments, and potentially more severe withdrawal symptoms when cannabis use is reduced or discontinued (Hindocha et al., 2021; Salinas et al., 2024). Considering these patterns, reliable tools are needed to assess withdrawal symptoms in populations where cannabis–tobacco co-use is frequent.

Standardized instruments are therefore essential for assessing cannabis withdrawal symptoms in both clinical and research settings (Livne et al., 2019). One of the most widely used tools in English-speaking settings is the Marijuana Withdrawal Checklist (also referred to as the Cannabis Withdrawal Checklist, CWC) (Budney et al., 1999). This 15-item self-report instrument captures a broad range of withdrawal symptoms and has demonstrated good reliability and sensitivity to change over time.

However, despite its widespread use, validated versions of the CWC are not currently available for Spanish-speaking populations. Cultural and linguistic adaptation is particularly relevant in Spain, where cannabis consumption patterns—particularly the frequent co-use with tobacco—may influence the expression of withdrawal symptoms.

Therefore, the present study aimed to culturally adapt and psychometrically validate the Cannabis Withdrawal Checklist (CWC) for Spanish-speaking individuals seeking treatment for their cannabis use disorder. Specifically, we evaluated the reliability, construct validity, and sensitivity to symptom change over time of the Spanish version of the scale at baseline and 2 weeks after treatment initiation.

## Materials and methods

### Study design

This study was designed as a preliminary psychometric validation of the CWC in the Spanish population. The study design followed key principles for evaluating patient-reported outcome measures, including the assessment of content validity, internal consistency, and structural validity, following the Consensus-based Standards for the selection of health Measurement Instruments (COSMIN) guidelines(Mokkink et al., 2016).

The scale validation was conducted within the multicenter, longitudinal DuCATA_GAM-CaT project, which examines cannabis withdrawal syndrome during treatment for CUD (Saura et al., 2024). The validation focused on withdrawal symptoms during the early phase of treatment, with assessments conducted at baseline and 2 weeks after the initiation of treatment for CUD (Budney and Hughes, 2006).

### Participant characteristics

Participants were recruited between April 2023 and December 2024 from 10 Catalan centers providing care for substance use disorders under the Substance Use Treatment Program (SUTP), where they received routine clinical care for CUD. The specific therapeutic approaches were determined by the treating clinicians according to standard practice at each center and were not standardized as part of the study protocol. The research team did not intervene in treatment decisions, as the study aimed to observe withdrawal symptoms in a real-world clinical context.

Inclusion criteria were as follows: (1) aged ≥18 years; (2) current cannabis use (with or without other substances); (3) a clinical diagnosis of CUD confirmed by a positive score (≥4) on the CAST and initiation of treatment for cannabis use disorder in one of the participating centers (Legleye et al., 2007); (4) access to a smartphone with WhatsApp Messenger; (5) willingness to participate in follow-up assessments over 6 weeks; and (6) provision of signed informed consent. Participants were enrolled at treatment entry for cannabis use disorder, which was considered the primary substance-related problem motivating treatment.

Exclusion criteria were as follows: (1) inability to read or understand Spanish; (2) moderate-to-severe cognitive impairment; and (3) severe or acute psychopathology. The presence of severe psychopathology or cognitive impairment was assessed during routine clinical evaluation at treatment entry by experienced clinicians at each participating center, as part of standard clinical care, and these assessments were not performed by the research team.

Although participants could report the use of other substances, the study focused on cannabis–tobacco co-use, the most common consumption pattern in Spain (OEDA, 2024), and other substances were not systematically assessed to minimize participant burden.

A sample size of approximately 100 participants was set, based on the recommendations of at least two participants per assessed item, which is acceptable for exploratory validation (Anthoine et al., 2014). Ninety-four individuals who met the inclusion criteria and agreed to participate were initially recruited. Withdrawal symptoms were assessed at two time points, corresponding to baseline and the second follow-up. The final analytic sample consisted of 82 participants, after excluding cases with incomplete or missing data.

### Original instrument and variables

The Cannabis (Marijuana) Withdrawal Checklist (CWC) (Budney et al., 1999) was selected for adaptation and validation in Spanish populations due to its simplicity and widespread clinical and research use. This instrument has been frequently used in clinical samples of individuals seeking treatment for cannabis use disorder, which aligns with the characteristics of our study population.

The CWC is a 15-item self-reported instrument designed to assess common symptoms of cannabis withdrawal. In this study, withdrawal symptoms were assessed exclusively through participants’ self-reported responses to the CWC, and no additional clinical assessment of withdrawal symptoms was performed. Each item is rated on a 4-point scale ranging from 0 (none) to 3 (severe), yielding a total score ranging from 0 to 45, with higher scores indicating greater withdrawal severity. In the original validation study, the scale showed good internal consistency (Cronbach’s alpha = 0.81 on day 3 of abstinence).

For this study, the scale was adapted for Spanish-speaking populations and renamed the Cannabis Withdrawal Checklist to reflect the term “cannabis,” which is more commonly used in Spain, and in recent scientific literature to refer broadly to cannabis products (including marijuana and hashish), while preserving the conceptual equivalence of the original items. Following the original instrument, we collected information on withdrawal symptoms that may occur within the first days of reducing or stopping cannabis use. These symptoms include the following:

- Shakiness: Shaking or trembling of the hands or body that is not due to physical exertion.
- Depressed mood: Persistent feelings of sadness, hopelessness, or low mood.
- Decreased appetite: Reduced desire or motivation to eat.
- Nausea: Stomach discomfort with an urge to vomit.
- Irritability: Heightened sensitivity to frustration or becoming easily annoyed
- Sleep difficulties: Problems falling or staying asleep.
- Sweating: Excessive perspiration without physical exertion.
- Craving for cannabis: Strong desire or urge to consume cannabis.
- Restlessness: Difficulty staying still or feeling physically agitated.
- Nervousness: Anxiety, uneasiness, or feeling on edge.
- Aggression: Tendency to react with hostility or verbal/physical aggression.
- Headaches: Persistent or intermittent pain in the head region.
- Stomach pain: Abdominal discomfort or cramps.
- Strange dreams: Unusual, vivid, or disturbing dreams.
- Increased anger: Elevated levels of irritability expressed as anger or rage.

In addition to withdrawal symptoms, sociodemographic variables, including age, sex (male and female), nationality (Spanish or other), education level (primary studies or less, secondary, and university), and employment status (employed, unemployed, student, and others), were collected. Patterns of cannabis and tobacco use were also recorded, including nicotine dependence assessed using the Fagerström Test for Nicotine Dependence (FTND) (Becoña and Vázquez, 1998), a widely used 6-item questionnaire with scores ranging from 0 to 10, where higher scores indicate greater nicotine dependence. The number of spliffs and cigarettes consumed was also recorded. At baseline, participants were also asked whether they had reduced their cannabis consumption within the previous 48 h through a self-reported yes/no question. Treatment-seeking status, referring to participants already enrolled in early-stage treatment for cannabis use disorder at the centers, was assessed at baseline and 2 weeks after treatment initiation to monitor changes over time.

### Translation and cultural adaptation

The scale was translated following standard cross-cultural adaptation procedures, including forward and backward translation conducted by the research team and clinicians with expertise in cannabis cessation. The translated version was then reviewed by the same team to ensure semantic and conceptual equivalence (Benavent et al., 2023). For cultural adaptation and psychometric analyses, the Spanish term “marihuana” (direct translation of “marijuana”) was replaced with the term “cannabis” throughout the instrument. The translated checklist of withdrawal symptoms is shown in the Supplementary Table S1.

### Psychometric assessment

Validation analyses were conducted, focusing on assessing the reliability and construct validity of the scale. Internal consistency was evaluated using Cronbach’s alpha coefficient, which ranges from 0 to 1. An alpha coefficient of >0.70 was considered indicative of acceptable internal consistency (Tavakol and Dennick, 2011).

Inter-item correlations were examined to assess potential redundancy among items and to ensure that all items measured the same underlying construct. Correlations were expected to be positive, with values below 0.80 considered acceptable to avoid redundancy between items, whereas low correlations (<0.20) were flagged for review.

Exploratory Factor Analysis (EFA) was conducted on baseline data to explore the underlying factor structure. The analysis used the minimum residuals extraction method with Varimax rotation. Factor retention was guided by multiple criteria, including parallel analysis, the Kaiser criterion (*Eigenvalues* > 1), and visual inspection of the scree plot (Costello and Osborne, 2005). Consistent with the exploratory nature of this preliminary validation study, the EFA was conducted using baseline data (*n* = 82), with a sample size within the range commonly reported in exploratory validation studies of patient-reported outcome measures (Anthoine et al., 2014).

Although confirmatory factor analysis (CFA) was initially considered to further evaluate the factorial structure, it was not performed due to the limited sample size, which could compromise the stability and interpretability of model fit indices.

Test–retest reliability was not calculated due to expected changes in cannabis use status over time. Instead, sensitivity to change was evaluated to examine whether the scale could detect differences in withdrawal symptom severity between baseline and 2 weeks after treatment initiation, using a paired-sample t-test (*α* = 0.05).

To explore whether withdrawal severity varied according to the patterns of substance use and participant characteristics, we fitted a linear mixed-effects model with a random intercept for participants to account for repeated observations over time. The mean withdrawal score was used as the dependent variable. Fixed effects included the number of cannabis–tobacco spliffs per day, the number of tobacco cigarettes per day, sex, and time of assessment (baseline vs. 2 weeks after treatment initiation). An interaction term between cannabis use (spliffs per day) and time of assessment was included to examine whether the association between cannabis use and withdrawal severity changed over time.

Data processing was conducted in Python version 3.8.5, and statistical analyses were performed in R version 4.3.2.

### Ethical approval

The study was approved by the Clinical Research Ethics Committee of Hospital Universitari de Bellvitge (Ref. PR328/19, Act 03/21, 11 February 2021), following earlier approval of the initial study protocol by the same committee (Act 22/19, 19 December 2019). All procedures were conducted in accordance with the Declaration of Helsinki and applicable national regulations.

## Results

### Sample description

The final sample included 82 participants, after excluding 12 individuals due to missing or incomplete data. A majority of participants were male (75.6%), with a mean age of 32.5 years (SD = 12.9). The majority were Spanish nationals (89.0%), and 66.2% had completed secondary education, while 23.8% reported primary education or less. Regarding employment status, 45.7% were employed, 42.0% were unemployed, 7.4% were students, and 4.9% had other situations (Supplementary Table S2).

At baseline, all participants reported daily tobacco use, with a mean Fagerström Test for nicotine dependence score of 4.2 (SD = 1.7). On average, participants smoked 7.3 tobacco cigarettes and 5.1 cannabis–tobacco spliffs per day. At 2 weeks after treatment initiation, slight reductions were observed (6.2 cigarettes and 4.6 spliffs per day), although these changes were not statistically significant.

### Descriptive analysis of cannabis withdrawal symptoms reported by the participants

The distribution of CWC item responses was examined at baseline and 2 weeks after cannabis treatment initiation. Descriptive statistics, including means, standard deviations, and 95% confidence intervals (CIs), are presented in Table 1. Average item scores were generally low (≤2 points), with no statistically significant differences between the scores at baseline and 2 weeks after treatment initiation for any item.

**Table 1:** Mean, standard deviation, and 95% confidence intervals for cannabis withdrawal symptoms at baseline and at 2 weeks of cessation treatment (Spanish sample, *n* = 82).

	Overall—baseline		Overall—At 2 weeks of treatment		
Withdrawal symptom	Mean (SD)	95%CI	Mean (SD)	95%CI	*p*-value*
Shakiness	0.49 (0.86)	0.32–0.67	0.40 (0.69)	0.18–0.61	0.801
Depression	1.39 (1.19)	1.15–1.63	1.53 (0.91)	1.26–1.81	0.402
Decreased appetite	1.13 (1.04)	0.92–1.34	1.05 (0.97)	0.75–1.35	0.690
Nausea	0.48 (0.88)	0.31–0.66	0.40 (0.88)	0.13–0.67	0.384
Irritability	1.25 (1.07)	1.03–1.46	1.12 (0.88)	0.85–1.39	0.613
Sleep difficulties	1.26 (1.08)	1.01–1.50	1.37 (1.16)	1.02–1.73	0.509
Sweating	0.86 (1.11)	0.63–1.08	0.81 (1.10)	0.48–1.15	0.856
Craving	1.61 (1.06)	1.40–1.82	1.65 (0.97)	1.35–1.95	0.823
Restlessness	1.47 (1.03)	1.27–1.68	1.47 (0.93)	1.18–1.75	0.977
Nervousness	1.72 (1.00)	1.52–1.92	1.53 (0.96)	1.24–1.83	0.305
Aggression	0.62 (0.86)	0.45–0.79	0.61 (0.90)	0.33–0.88	0.782
Headaches	0.80 (1.08)	0.59–1.02	0.58 (0.85)	0.32–0.84	0.378
Stomach pains	0.71 (1.00)	0.51–0.91	0.65 (0.87)	0.38–0.92	1.000
Strange dreams	0.76 (1.02)	0.56–0.97	0.98 (1.20)	0.61–1.35	0.485
Increased anger	0.80 (0.94)	0.61–0.99	0.56 (0.85)	0.30–0.82	0.114

SD, standard deviation, CI, confidence interval.

*Mann–Whitney U-test.

At baseline, the mean of all individual withdrawal symptom scores (ranging from 0 = none to 3 = severe) was 0.94. The total withdrawal score (sum of all 15 items, range 0–45) was 15.4 (SD = 10.5) at baseline and 14.7 (SD = 8.8) at 2 weeks after treatment initiation. Individual symptom scores are presented in Table 1. A majority of the participants reported experiencing multiple withdrawal symptoms at baseline (around 84% of the sample), and symptom scores remained relatively stable over the 2-week follow-up period, with no statistically significant changes observed for any individual symptom.

### Reliability of the instrument

The Spanish version of the CWC demonstrated high internal consistency, with Cronbach’s alpha values of 0.918 (95% CI, 0.889–0.940) at baseline and 0.885 (95% CI, 0.781–0.936) at 2 weeks after treatment initiation, and *p* = 0.221 (Feldt’s test), indicating adequate temporal stability. Item-by-item deletion analyses showed no improvement in Cronbach’s alpha values, suggesting that all items contributed meaningfully to the scale (Table 2).

**Table 2:** Internal consistency (Cronbach’s alpha) of the Spanish version of the Cannabis Withdrawal Checklist, with item-by-item deletion analyses at baseline and at 2 weeks of treatment.

Item	Baseline	At 2 weeks of treatment	*p*-value
	Alpha^1^ (95%CI)	Alpha^1^ (95%CI)	Feldt test
**All items considered**	**0.918 (0.889–0.940)**	**0.885 (0.781–0.936)**	0.221
Deleting one single item			
Depression	0.910	0.872	
Aggression	0.911	0.880	
Increased anger	0.910	0.875	
Nervousness	0.907	0.869	
Restlessness	0.909	0.871	
Craving	0.915	0.885	
Irritability	0.910	0.878	
Decreased appetite	0.913	0.875	
Sweating	0.913	0.881	
Strange dreams	0.918	0.890	
Shakiness	0.912	0.878	
Nausea	0.913	0.881	
Stomach pains	0.914	0.879	
Sleep difficulties	0.914	0.875	
Headaches	0.914	0.876	

^1^Cronbach’s alpha.

CI, confidence interval.

Bold values indicate overall Cronbach’s alpha values.

All inter-item correlations were positive and below the threshold for multicollinearity (*r* < 0.80), confirming that items clustered around a common construct without redundancy (Supplementary Table S3).

### Internal structure validity

#### Exploratory factor analysis (EFA)

Sampling adequacy was confirmed by a Kaiser–Meyer–Olkin (KMO) index of 0.860, indicating excellent suitability for factor analysis. Bartlett’s test of sphericity was significant (*p* < 0.001), confirming that correlations between items justified factor extraction. Only baseline data were used for the EFA, and missing values were handled via listwise deletion. Parallel analysis and the Kaiser criterion supported a one-factor solution, which explained approximately 60% of total variance (Supplementary Table S4). All items loaded significantly onto this factor (Table 3). The model, however, did not fit optimally (χ^2^ = 295.4, *p* < 0.001).

**Table 3:** Exploratory factor analysis (EFA) of the Spanish Cannabis Withdrawal Checklist at baseline (*n* = 82).

Parameter	Estimate^1^	Standard error	*z*-value	*p*-value^2^
Latent variables				
Depression	1.000 (Ref.)	-	-	-
Aggression	0.677	0.102	6.643	<0.001
Increased anger	0.777	0.113	6.872	<0.001
Nervousness	0.970	0.122	7.977	<0.001
Restlessness	0.945	0.126	7.510	<0.001
Craving	0.673	0.129	5.215	<0.001
Irritability	0.912	0.130	7.018	<0.001
Decreased appetite	0.742	0.126	5.899	<0.001
Sweating	0.788	0.135	5.829	<0.001
Strange dreams	0.608	0.126	4.831	<0.001
Shakiness	0.672	0.106	6.343	<0.001
Nausea	0.563	0.105	5.382	<0.001
Stomach pains	0.651	0.123	5.284	<0.001
Sleep difficulties	0.860	0.149	5.787	<0.001
Headaches	0.783	0.133	5.904	<0.001
Residual variances				
Depressed	0.658	0.105	6.280	<0.001
Aggression	0.353	0.055	6.363	<0.001
Increased anger	0.407	0.065	6.294	<0.001
Nervousness	0.305	0.054	5.683	<0.001
Restlessness	0.405	0.067	6.019	<0.001
Craving	0.757	0.114	6.629	<0.001
Irritability	0.514	0.082	6.243	<0.001
Decreased appetite	0.638	0.098	6.529	<0.001
Sweating	0.745	0.114	6.541	<0.001
Strange dreams	0.761	0.114	6.672	<0.001
Shakiness	0.410	0.064	6.440	<0.001
Nausea	0.485	0.073	6.608	<0.001
Stomach pains	0.682	0.103	6.621	<0.001
Sleep difficulties	0.910	0.139	6.548	<0.001
Headaches	0.708	0.108	6.528	<0.001
Factor 1	**0.668**	0.168	3.975	<0.001
Model information				
Model fit	Chi-square = 295.37; *p*-value < 0.001; degrees of freedom = 104			
Information Criteria	AIC = 3473.06; BIC = 3551.19			
Residuals	Root mean square error of approximation (RMSEA) = 0.140; *p*-value < 0.001			
Sample size	82			

^1^Estimate = factor loading, indicating the strength of association between each item and the latent factor.

^2^*P*-value indicates whether the loading is significantly different from zero; a significant p-value suggests the item contributes meaningfully to the factor.

Bold values indicate the variance explained by the factor.

### Mixed-effects analysis: relationship between withdrawal syndromes, use, and time of assessment

Although the primary aim of this study was the validation of the withdrawal scale, we additionally explored whether withdrawal severity varied according to cannabis and tobacco use, sex, and time of assessment using a linear mixed-effects model with a random intercept for participants. The intercept was significant (*β* = 8.10, *p* < 0.001), indicating substantial baseline withdrawal severity in the reference group (male participants, baseline assessment, and average number of tobacco cigarettes).

Cannabis use intensity, measured as the number of spliffs smoked per day, was positively associated with withdrawal severity (*β* = 0.824, *p* = 0.005), and female participants showed higher withdrawal scores than male participants (*β* = 6.53, *p* = 0.004). Time (two weeks after treatment initiation vs. baseline) and the number of tobacco cigarettes were not significantly associated with withdrawal severity (*β* = 0.075, *p* = 0.975; *β* = 0.196, *p* = 0.125, respectively), and the interaction between time and cannabis use was also non-significant (*β* = −0.060, *p* = 0.890), suggesting that the effect of cannabis use on withdrawal did not change over the 2-week period (Table 4).

**Table 4:** Linear mixed model overall withdrawal symptoms for 48 days follow-up.

Predictors	Estimates	Standard error	*p*-value
Intercept	8.095	2.112	**<0.001**
Time: Week 2	0.075	2.388	0.975
Number of spliffs	0.824	0.291	**0.005**
Number of tobacco cigarettes	0.196	0.127	0.125
Sex: female	6.528	2.216	**0.004**
Time* Number of spliffs	−0.06	0.427	0.890
Random effects			
R^2^ marginal	0.157		
R^2^ conditional	0.718		
ICC	0.665		
N observation	120		
N participants	81		

*Wald test.

Bold values indicate statistically significant results (*p* < 0.05).

The random intercept indicated important individual differences between participants, with an intraclass correlation coefficient (ICC) of 0.665. The model explained 15.7% of the variance with fixed effects alone (marginal R^2^) and 71.8% when including both fixed and random effects (conditional R^2^), highlighting the contribution of individual differences to withdrawal severity.

## Discussion

This study assessed the psychometric properties of the CWC Scale in a Spanish-speaking sample, focusing on cannabis withdrawal symptoms and their association with cannabis use patterns. Previous instruments, such as the Marijuana Withdrawal Checklist (Budney et al., 1999) and the Cannabis Withdrawal Scale (Allsop et al., 2012), were validated primarily in English-speaking populations. Therefore, this study provides a first step toward addressing the gap by offering a culturally adapted Spanish version.

### Withdrawal symptom severity

The mean of all item scores across the 15 symptoms was 1.02, indicating low-to-moderate symptom intensity, while confirming the presence of cannabis withdrawal symptoms in our sample. Comparable scores were reported in an American sample (Agrawal et al., 2008), with an average score of 1.37. Although slightly higher values were observed in that study, the overall pattern of withdrawal severity is consistent with findings reported in previous clinical samples. In the original validation study, a majority of the participants reported experiencing four or more withdrawal symptoms, as observed in our sample, where 85% of participants reported experiencing withdrawal symptoms (Budney et al., 1999). These results suggest that, despite moderate withdrawal symptom intensity, the scale effectively captures clinically relevant withdrawal experiences.

### Psychometric properties: reliability

The Spanish CWC demonstrated high internal consistency at both assessment points, aligning with the original validation of the Marijuana (Cannabis) Withdrawal Checklist [*α* = 0.89; Budney et al. (1999)]. Stability from baseline (*α* = 0.92) to 2 weeks after treatment initiation (*α* = 0.89) further reinforces its temporal reliability. Item-by-item deletion analyses did not improve internal consistency, suggesting that all items contributed meaningfully to the construct.

### Factor structure and validity

Exploratory factor analysis supported a single-factor solution explaining 60% of the variance, consistent with the original validation study of the Marijuana Withdrawal Checklist (Budney et al., 1999). These findings suggest that the withdrawal symptoms captured by the scale cluster around a common underlying construct in this clinical population.

This result differs from the two-factor structure proposed by Hasin et al. (2008) in the general population sample, which distinguishes between “weakness” and “anxiety/depression” domains (Hasin et al., 2008). Differences in sample characteristics, particularly the inclusion of treatment-seeking individuals with cannabis use disorder, may partly explain this discrepancy.

### Inter-item correlation

The pattern of inter-item correlations indicated that cannabis withdrawal symptoms clustered coherently within a single construct without redundancy, supporting the hypothesis of a unidimensional structure. Similar findings have been reported in previous studies, where withdrawal symptoms exhibit consistent yet heterogeneous covariation, making it difficult to group them into clearly defined factors (Allsop et al., 2012; Hasin et al., 2008). The consistency in the direction of correlations suggests that the items converge adequately to capture a common underlying phenomenon.

### Temporal change and relationship with cannabis use

No significant differences were observed in total withdrawal scores between baseline and 2 weeks after treatment initiation (mean = 15.4, SD = 10.5 at baseline; mean = 14.7, SD = 8.8 at 2 weeks after treatment initiation), which may reflect the relatively small changes in cannabis use observed during the early phase of treatment. Withdrawal was self-reported and not biochemically verified (Hasin et al., 2013). Cessation programs focused primarily on cannabis as the target substance. Withdrawal severity was positively associated with the daily number of spliffs, reflecting a dose–response relationship in which higher cannabis consumption corresponded to more severe withdrawal symptoms (Budney and Hughes, 2006; Livne et al., 2019), supporting the scale’s sensitivity to variations in consumption patterns.

### Cannabis–tobacco co-use

All participants were daily users of tobacco and consumed cannabis primarily as spliffs (i.e., mixed with tobacco), reflecting common patterns in Spain and Europe (Blasco et al., 2024; Cooper and Haney, 2009), where pure cannabis use is relatively uncommon. While nicotine dependence may contribute to some withdrawal experiences (Agrawal et al., 2008), our analyses were not designed to disentangle the specific contributions of cannabis and nicotine use to withdrawal symptoms. The Spanish CWC demonstrated robust psychometric properties even within this context of co-use. Moreover, cannabinoid withdrawal affects brain reward pathways in ways similar to those of other addictive substances (Budney et al., 1999), consistent with the irritability and discomfort reported by our participants. This finding suggests that the instrument is suitable for real-world clinical populations in Spain. Future studies could include participants who do not use tobacco to clarify the distinct contributions of cannabis versus nicotine withdrawal.

### Limitations and strengths

This study has several limitations. First, the modest sample size may limit the stability and generalizability of the exploratory factorial results, and therefore, the findings should be interpreted as preliminary. Second, reliance on self-reported data and the absence of an external gold standard limit the certainty that reported symptoms fully reflect withdrawal rather than continued use. Third, although participants were enrolled because they were initiating treatment for cannabis use disorder, the use of substances other than cannabis and tobacco was not systematically assessed. Therefore, the contribution of other substances to the reported symptoms cannot be completely ruled out. Fourth, all participants were co-users of cannabis and tobacco; future studies should consider including a control group of participants who do not use tobacco to examine whether co-use affects withdrawal symptomatology. Finally, although replacing the term “marijuana” with “cannabis” improves cultural relevance, it may affect comparability with studies using the original terminology.

Despite these limitations, the study has important strengths. The longitudinal design and the application of robust psychometric and factor analytic methods support the internal validity of the findings, allowing reliable tracking of withdrawal symptom changes over time, which is particularly relevant in clinical contexts. The extent of missing data (36%) at 2 weeks after treatment initiation may limit the generalizability of the findings and should be considered when interpreting the results.

### Clinical applications and future directions

The Spanish CWC is a practical and accessible tool for monitoring cannabis withdrawal symptoms in Spanish-speaking populations. Its clarity and brevity make it suitable for individuals with lower educational levels, supporting equitable assessment across diverse socioeconomic backgrounds. Clinicians can use the scale to identify withdrawal severity, track changes over time, and tailor interventions according to patients’ needs, facilitating treatment planning and decision-making in outpatient or community-based settings. The adapted checklist used in this study is provided in the Supplementary Table S1 to facilitate its use in future research and clinical settings.

In addition, the tool’s usability supports its integration into real-world clinical practice, enabling rapid evaluation without requiring extensive training. Future research should focus on expanding validation to diverse populations, including vulnerable groups and users of other substances and clinical settings, assessing its implementation in routine care, and exploring its potential to improve equitable access to treatment and evidence-based interventions. Future studies should also include objective verification of abstinence and larger and more diverse samples to strengthen psychometric evidence and should further explore the differential effects of cannabis and nicotine use on withdrawal experiences. In addition, when relevant, future studies should examine differences using a control group of participants who do not use tobacco to clarify the influence of co-use.

## Conclusion

The Spanish CWC demonstrates strong internal consistency and effectively captures clinically relevant withdrawal symptoms, with severity correlating with cannabis use. Exploratory analyses supported a unidimensional structure, although confirmatory results were less conclusive. The high prevalence of cannabis–tobacco co-use reflects real-world patterns in Spain, and while it may complicate interpretation, the CWC nevertheless demonstrated strong psychometric properties in this context. Overall, these findings indicate that the Spanish CWC is a reliable, valid, and practical tool for assessing cannabis withdrawal symptoms in Spanish-speaking clinical and research populations.

## Data Availability

The raw data supporting the conclusions of this article will be made available by the authors, without undue reservation.
