# Supplementary material for: Psychometric validation of the Cannabis Withdrawal Checklist in a Spanish sample with cannabis use disorder
Source: Front Psychol. 2026 May 15;17:1744004. doi: 10.3389/fpsyg.2026.1744004 (PMC13219039; doi:10.3389/fpsyg.2026.1744004)
Supplement: Supplementary file 1 [file Table_1.docx]

**Psychometric validation of Cannabis Withdrawal Checklist in a Spanish sample with cannabis use disorder**

-Supplementary Tables-

Supplementary Table S1. Spanish Translation of the 15-Item Marihuana (Cannabis) Withdrawal Checklist Scale and Scoring.

| **Escala de síntomas de abstinencia de cannabis [Marijuana Withdrawal Checklist]** | | | | |
| --- | --- | --- | --- | --- |
| **Síntomas [Symptoms]** | **Ninguno [None]** | **Leve**  **[Mild]** | **Moderado [Moderate]** | **Grave**  **[Severe]** |
| 1) Temblores [Shakiness] | 0 | 1 | 2 | 3 |
| 2) Estado de ánimo deprimido [Depression mood] | 0 | 1 | 2 | 3 |
| 3) Pérdida de apetito [Decreased Appetite] | 0 | 1 | 2 | 3 |
| 4) Náuseas [Nausea] | 0 | 1 | 2 | 3 |
| 5) Irritabilidad [Irritability] | 0 | 1 | 2 | 3 |
| 6) Dificultad para dormir [Sleep difficulty] | 0 | 1 | 2 | 3 |
| 7) Sudoración [Sweating] | 0 | 1 | 2 | 3 |
| 8) Deseo de consumir marihuana [Craving] | 0 | 1 | 2 | 3 |
| 9) Inquietud [Restlessness] | 0 | 1 | 2 | 3 |
| 10) Nerviosismo [Nervousness] | 0 | 1 | 2 | 3 |
| 11) Aumento de la agresividad [Agression] | 0 | 1 | 2 | 3 |
| 12) Dolores de cabeza [Headaches] | 0 | 1 | 2 | 3 |
| 13) Dolores estomacales [Stomach pain] | 0 | 1 | 2 | 3 |
| 14) Sueños extraños [Strange dreams] | 0 | 1 | 2 | 3 |
| 15) Aumento de la ira [Increased anger] | 0 | 1 | 2 | 3 |

Supplementary Table S2. Demographic and clinical characteristics of participants in the Spanish sample receiving treatment for problematic cannabis use (baseline, n = 82).

|  | | | |
| --- | --- | --- | --- |
|  |  | N (%) | 95% CI |
|  |  |  |  |
| **Sociodemographic Characteristics** |  |  |  |
| **Sex** |  |  |  |
| Male |  | 62 (75.6) | 64.9-84.4 |
| Female |  | 20 (24.4) | 15.6-35.1 |
|  |  |  |  |
| **Age (mean, [sd])** |  | 32.5 [12.9] | 29.8-35.3 |
|  |  |  |  |
| **Nationality** |  |  |  |
| Spanish |  | 73 (89.0) | 80.2-94.9 |
| Other |  | 9 (11.0) | 5.1-19.8 |
|  |  |  |  |
| **Educational level** |  |  |  |
| Primary studies or less |  | 19 (23.8) | 15.2-34.8 |
| Secondary education |  | 53 (66.2) | 53.2-74.7 |
| University |  | 8 (10.0) | 4.6-18.8 |
|  |  |  |  |
| **Employment situation** |  |  |  |
| Employed |  | 37 (45.7) | 34.2-56.5 |
| Unemployed |  | 34 (42.0) | 30.9-52.9 |
| Student |  | 6 (7.4) | 3.0-15.8 |
| Others |  | 4 (4.9) | 1.6-12.7 |
|  |  |  |  |
|  |  |  |  |
| **Consumption Characteristics** |  |  |  |
| **Tobacco daily -baseline** |  |  |  |
| Yes |  | 82 (100.0) | 94.4-100.0 |
| **Nicotine dependence at baseline**  **(mean, [sd])** |  | 4.21 [1.70] | 3.85-4.57 |
|  |  |  |  |
| **Number of tobacco cigarettes per day**  **(mean, [sd])** |  |  |  |
| Baseline |  | 7.3 [7.0] | 5.8-8.9 |
| Week 2 |  | 6.2 [6.1] | 4.9-7.5 |
|  |  |  |  |
| **Number of spliffs per day**  **(mean, [sd])** |  |  |  |
| Baseline |  | 5.1 [3.0] | 4.5-5.7 |
| Week 2 |  | 4.6 [2.7] | 4.0-5.2 |
| CI: confidence interval  Sd= Standard deviation |  |  |  |

Supplementary Table S3. Correlation of withdrawal symptoms at baseline and at two weeks of treatment^1^

| Baseline | Depression | Aggression | Increased Anger | Nervousness | Restlessness | Craving | Irritability | Decreased Appetite | Sweating | Strange Dreams | Shakiness | Nausea | Stomach Pains | Sleep Difficulty | Headaches |
| --- | --- | --- | --- | --- | --- | --- | --- | --- | --- | --- | --- | --- | --- | --- | --- |
| Depression | 1.00 |  |  |  |  |  |  |  |  |  |  |  |  |  |  |
| Aggression | 0.43 | 1.00 |  |  |  |  |  |  |  |  |  |  |  |  |  |
| Increased Anger | 0.41 | 0.83 | 1.00 |  |  |  |  |  |  |  |  |  |  |  |  |
| Nervousness | 0.69 | 0.52 | 0.53 | 1.00 |  |  |  |  |  |  |  |  |  |  |  |
| Restlessness | 0.60 | 0.49 | 0.48 | 0.79 | 1.00 |  |  |  |  |  |  |  |  |  |  |
| Craving | 0.45 | 0.36 | 0.31 | 0.49 | 0.52 | 1.00 |  |  |  |  |  |  |  |  |  |
| Irritability | 0.56 | 0.62 | 0.70 | 0.62 | 0.57 | 0.33 | 1.00 |  |  |  |  |  |  |  |  |
| Decreased Appetite | 0.53 | 0.39 | 0.40 | 0.54 | 0.36 | 0.37 | 0.35 | 1.00 |  |  |  |  |  |  |  |
| Sweating | 0.49 | 0.47 | 0.41 | 0.53 | 0.38 | 0.30 | 0.40 | 0.33 | 1.00 |  |  |  |  |  |  |
| Strange Dreams | 0.20 | 0.37 | 0.47 | 0.38 | 0.46 | 0.31 | 0.34 | 0.44 | 0.32 | 1.00 |  |  |  |  |  |
| Shakiness | 0.52 | 0.49 | 0.60 | 0.48 | 0.35 | 0.26 | 0.34 | 0.47 | 0.55 | 0.36 | 1.00 |  |  |  |  |
| Nausea | 0.45 | 0.43 | 0.47 | 0.39 | 0.52 | 0.20 | 0.44 | 0.54 | 0.39 | 0.31 | 0.46 | 1.00 |  |  |  |
| Stomach Pains | 0.37 | 0.34 | 0.39 | 0.44 | 0.41 | 0.32 | 0.36 | 0.34 | 0.43 | 0.40 | 0.35 | 0.32 | 1.00 |  |  |
| Sleep Difficulty | 0.46 | 0.36 | 0.37 | 0.53 | 0.56 | 0.43 | 0.36 | 0.20 | 0.43 | 0.23 | 0.42 | 0.30 | 0.56 | 1.00 |  |
| Headaches | 0.40 | 0.41 | 0.39 | 0.43 | 0.51 | 0.44 | 0.55 | 0.39 | 0.26 | 0.40 | 0.63 | 0.54 | 0.24 | 0.40 | 1.00 |

| At two weeks of treatment | Depression | Aggression | Increased  Anger | Nervousness | Restlessness | Craving | Irritability | Decreased  Appetite | Sweating | Strange  Dreams | Shakiness | Nausea | Stomach  Pains | Sleep  Difficulty | Headaches |
| --- | --- | --- | --- | --- | --- | --- | --- | --- | --- | --- | --- | --- | --- | --- | --- |
| Depression | 1.00 |  |  |  |  |  |  |  |  |  |  |  |  |  |  |
| Aggression | 0.21 | 1.00 |  |  |  |  |  |  |  |  |  |  |  |  |  |
| Increased Anger | 0.31 | 0.85 | 1.00 |  |  |  |  |  |  |  |  |  |  |  |  |
| Nervousness | 0.54 | 0.52 | 0.53 | 1.00 |  |  |  |  |  |  |  |  |  |  |  |
| Restlessness | 0.68 | 0.36 | 0.35 | 0.78 | 1.00 |  |  |  |  |  |  |  |  |  |  |
| Craving | 0.49 | 0.20 | 0.27 | 0.54 | 0.50 | 1.00 |  |  |  |  |  |  |  |  |  |
| Irritability | 0.46 | 0.48 | 0.67 | 0.55 | 0.40 | 0.27 | 1.00 |  |  |  |  |  |  |  |  |
| Decreased Appetite | 0.54 | 0.32 | 0.37 | 0.53 | 0.45 | 0.17 | 0.35 | 1.00 |  |  |  |  |  |  |  |
| Sweating | 0.44 | 0.21 | 0.29 | 0.39 | 0.25 | 0.00 | 0.37 | 0.32 | 1.00 |  |  |  |  |  |  |
| Strange Dreams | 0.20 | 0.36 | 0.36 | 0.18 | 0.20 | 0.11 | 0.09 | 0.22 | 0.18 | 1.00 |  |  |  |  |  |
| Shakiness | 0.52 | 0.20 | 0.42 | 0.35 | 0.37 | 0.21 | 0.43 | 0.36 | 0.54 | 0.25 | 1.00 |  |  |  |  |
| Nausea | 0.33 | 0.11 | 0.11 | 0.31 | 0.38 | 0.08 | 0.16 | 0.56 | 0.38 | 0.49 | 0.61 | 1.00 |  |  |  |
| Stomach Pains | 0.36 | 0.10 | 0.24 | 0.43 | 0.47 | 0.19 | 0.09 | 0.41 | 0.20 | 0.24 | 0.29 | 0.38 | 1.00 |  |  |
| Sleep Difficulty | 0.42 | 0.30 | 0.29 | 0.46 | 0.54 | 0.25 | 0.26 | 0.39 | 0.47 | 0.00 | 0.35 | 0.66 | 0.67 | 1.00 |  |
| Headaches | 0.57 | 0.21 | 0.33 | 0.34 | 0.40 | 0.25 | 0.19 | 0.43 | 0.32 | 0.18 | 0.32 | 0.79 | 0.32 | 0.28 | 1.00 |

Supplementary Table S4. Parallel analysis of the Spanish Cannabis Withdrawal Checklist at baseline, estimating the optimal number of factors.

| Factor | Explained variation | Accumulated variation |
| --- | --- | --- |
| **1** | **0.583** | **0.583** |
| 2 | 0.089 | 0.672 |
| 3 | 0.080 | 0.752 |
| 4 | 0.063 | 0.815 |
| 5 | 0.048 | 0.863 |
| 6 | 0.038 | 0.901 |
| 7 | 0.029 | 0.930 |
| 8 | 0.026 | 0.956 |
| 9 | 0.020 | 0.976 |
| 10 | 0.009 | 0.986 |
| 11 | 0.008 | 0.994 |
| 12 | 0.005 | 0.999 |
| 13 | 0.001 | 1.000 |
| 14 | 0.000 | 1.000 |
| 15 | 0.000 | 1.000 |
| Kaiser, Mayer, Okin index (KMO) = 0.860 | | |
| Bartlett's test < 0.001 | |  |
